# Vienna Letter

**Published:** 1882-05

**Authors:** W. T. Belfield

**Affiliations:** Vienna


### Foreign Correspondence.

ARTICLE XII.

VIENNA, AHRIL 2, 1882.

EDITORS CHICAGO MEDICAL JOURNAL AND EXAMINER:— The question as to the value of iodoform in surgery (or rather as to the dangers attending its use, for in regard to its good qualities there is no essential difference of opinion) is still the subject of animated discussion. The deductions of König, as a commentary to his report of disastrous cases in recent numbers of the *Centralblatt für Chirurgie*, have called forth energetic protest from god-father Mosetig Moorhof. He says: "I have never had a case of intoxication from iodoform, although I have used iodoform dressings almost exclusively during the last four years, in the treatment of some 3,000 in-patients and about 4,000 out-patients." He explains this fortunate immunity by the facts, 1, that the iodoform is never used in large quantities (70 grams is his maximum, I believe); 2, that it is never kept in contact with the wounded surface *under pressure;* 3, that the dressing is changed only at long intervals; 4, that in changing the bandage the wound is never syringed out in order to apply fresh iodoform, because, as is known, absorption proceeds more rapidly in granulating than in fresh wounds; 5, because the iodoform is used alone, *i. e.*, without any other antiseptic. He lays great stress upon this latter measure, and denounces especially the (in Germany almost universal) combination of carbolic acid with iodoform. He refers to several cases of reported intoxication, in one of which the reporter (Höftman) says: "The scanty urine, containing large quantities of carbolic acid, first showed the presence of iodoform on the third day, after disappearance of the carbolic acid." In another (König's): "The urine is almost black, but contains no iodine salts." Now, inasmuch as Mosetig, who does not use carbolic acid, always finds iodine in the *first* urine after an iodoform dressing; and inasmuch as carbolic acid is known to cause irritation, even inflammation of the kidneys, Mosetig is inclined to ascribe the retention of the iodine in the blood, and hence the intoxication, to the use of carbolic acid. He urges, therefore, the exclusive use of the one or the other antiseptic.

Winiwarter, in Liège, a former assistant of Billroth, has recently appeared among the champions of iodoform. He has secured all of the good and none of the bad results ascribed to the dressing, having never seen a case of intoxication. He irrigates the wound with carbolized water, and usually drains. He paints superficial wounds with a solution of iodoform in collodion.

In another of the large surgical clinics in Vienna (Prof. Dittel's) iodoform has been the staple dressing for the past year, without having caused any decided symptoms of intoxication.

Reports of pylorus resection appear not infrequently in the journals. Nicolaysen recently made one—death in twelve hours from collapse. Lauenstein, in Hamburg, recently experienced a hitherto unknown complication of this operation. The omentum was attached over almost the entire posterior surface of the stomach, and had to be largely separated from this organ, with ligature of its vessels. At the autopsy, several days later, there was found gangrene of the transverse colon. Lauenstein is inclined to assume a relation of cause and effect between the extensive detachment of omentum and the gangrene.

Rydygier, who made the first pylorus resection for cancer, recently made the first similar operation for stricture of the pylorus, with extensive dilatation of the stomach, following an obstinate gastric ulcer. The excised pylorus permitted the passage only of a number nine catheter. Patient's temperature never exceeded 39.3 C., and she was discharged cured six weeks after the operation. *Volkmann's Journal* says this is the first, "and it is to be hoped the last" performance of the operation for this pathological condition.

The recent discussion in the Berlin Medical Society of the results of nerve-stretching in general, but particularly of sciatic stretching in cases of tabes dorsalis, has drawn general attention to the subject. Some months ago, Benedikt, of Vienna, published some startling statements as the results of his experience in twenty-five cases. Among them was the following: A classical case of tabes, patient being scarcely able to hobble with the support of two sticks. The girdle sensation, rigidity of pupils, constipation, paralysis of bladder, impotence, absence of patellar reflex, paraesthesia, were the prominent symptoms. The right sciatic was severely stretched. Two weeks later patient walked almost without staggering, with closed eyes and without support of any sort. At the end of two months after the operation, the above mentioned symptoms (except abnormality of pupils and of patellar reflex) had almost or entirely disappeared, and the patient walked a mile easily.

Some months later he entered Bamberger's wards suffering from endo- and pericarditis. Here the absence of essential spinal symptoms was determined—even the patella reflex was now present. The autopsy revealed gray degeneration of the posterior columns throughout the medulla. Benedikt obtained some pieces of the spinal cord, and announced that there were evidences of regeneration of the diseased nerve-fibers.

This communication was received with general incredulity; in some quarters even with ridicule. It seems, however, to have secured at least one important result, namely, the discussion of the subject by competent men.

Schultze, of Heidelberg, published a case of undoubted tabes dorsalis, in which all symptoms except slight paresis of the bladder and the absence of the tendon reflexes disappeared during treatment by Erb with the continued current. This improvement continued unimpaired until death, several years later, from an intercurrent affection. The autopsy showed the usual degeneration in the posterior columns.

This and similar cases prove the possibility of a clinical recovery from this affection, perhaps, as Senator suggested in the Berlin society, from a vicarious assumption by the gray matter of the duties of the posterior columns.

A most important contribution to the subject is Langenbech's report on 100 operations, mostly stretching of the sciatic and crural nerves in cases of tabes. He observes that inasmuch as tabetic patients lose ground by keeping the recumbent posture, old, feeble, and fat persons are, for this, among many other reasons, less eligible for the operation. His experience does not confirm the rather natural *a priori* assumption that the chances of success are inversely proportioned to the stage of the disease; he has had most satisfactory results in some very advanced, and has utterly failed to improve in some very early cases. He frankly states that in the majority of cases he has seen either no benefit whatever or a merely temporary improvement follow the operation. Yet in many—some of which he reports minutely—there was a very marked and permanent improvement in all the troublesome symptoms, amounting in several cases to a practical cure.

At first he stretched with but little force and had good results; but becoming bolder, he used considerable force; in these cases he found that although immediate improvement occurred, yet in two to four months atrophy of the thigh muscles occurred. To avoid this, which he was inclined to attribute to tearing of small muscular branches, he in subsequent cases exposed the nerve at a part which gives off no branches—under the belly of the biceps. Since this change, he has never observed any considerable atrophy or paralysis of the muscles after the operation.

To avoid, so far as possible, the formation of cicatricial tissues around the nerve, the presence of which often caused unpleasant and painful after-effects, Langenbuch *cuts* all tissues down to the nerve itself. He then puts two carbolized sponges between the nerve and the fingers, to avoid local crushing. The tension must always be exerted on the central end only. He gives no directions as to the amount of force to be used, but warns against raising the patient, or even the leg, from the table by means of the nerve—a proceeding which Billroth in at least one case adopted. He insists upon antisepsis, by either carbolic acid or iodoform.

Upon rising from the bed after the immediate effects of the operation, the patient must begin systematic exercise.

Stretching of the crural nerve is more difficult, on account of its early division into branches; yet this operation has been, in

Langenbuch's hands, very serviceable in relieving the lightning pains of tabes. He advises that the division of the skin shall be limited to the integument of the abdomen, ending at Poupart's ligament.

In the discussion which followed the reading of this paper in the Berlin society, Westphal related his experience, taking a decidedly pessimistic position. Has observed some fifteen cases. In the first (1877) the result of stretching both crural nerves in a case of tabes was paralysis of both legs, bladder and rectum; severe bed-sores; transverse myelitis. In the other cases, the result was either *nil*, or a slight, transient diminution of pain. Thinks that these temporary improvements are due to purely psychical, not anatomical, processes. Westphal referred, further, to the celebrated original case of nerve-stretching by Nussbaum, for the relief of intercostal neuralgia. Some time after the publication of the asserted cure, Westphal learned, from a personal interview with the patient, that there was absolutely no improvement.

Bardeleben has performed the operation a number of times—once with complete recovery for a few weeks—subsequently relapse to worse than before. He performs the operation willingly, because " it's a good thing for the students to see."

Küster has observed two cases; one of temporary, one of permanent, improvement.

Senator is inclined to encourage the operation, although results to date have not been brilliant. Is convinced that recovery can and does occur, as in Erb's case (referred to above).

W. T. BELFIELD.

---

WE have just received from John H. Rauch, M. D., Secretary State Board of Health, a quantity of postal card records of vaccinations, which may be had on application at this office. We sincerely hope that the profession throughout the State will cooperate with the State Board of Health in their effort to have a complete record of vaccination.

